# Assessment of nutritional literacy and its association with glycemic control and obesity in type 2 diabetic patients in Bahrain

**DOI:** 10.3389/fnut.2026.1889663

**Published:** 2026-09-14

**Authors:** Ahmed Almansoor, Sara Alansari, May Almudhahka, Fatema Algaoud, Mahiyah Hesham, Shaikha Alshamsi, Mohamed Buhazza, May Alghatam, Alya Alhammadi, Hasan Albuainain, Fatima Alshinawi, Maryam Alnefaiei, Amer Almarabheh, Jamil Ahmed

**Affiliations:** Department of Family and Community Medicine College of Medicine and Health Sciences, Arabian Gulf University, Manama, Bahrain

**Keywords:** Bahrain, glycemic control, nutritional literacy, obesity, type 2 diabetes

## Abstract

**Introduction:**

Limited evidence exists on the association between nutritional literacy, glycemic control, and obesity among patients with type 2 diabetes mellitus (T2DM) in Bahrain. This study assessed nutritional literacy and its association with glycemic control and obesity among adults with T2DM.

**Methods:**

A cross-sectional study was conducted among 364 adults with T2DM recruited using consecutive sampling from public primary healthcare centers across Bahrain between July and August 2024. Data were collected through face-to-face interviews using a pilot-tested structured questionnaire administered by trained researchers (Cronbach’s α = 0.78). Glycemic control (HbA1c) and body mass index (BMI) were obtained from medical records. Data were analyzed using descriptive statistics, Pearson’s chi-square test, and multivariable logistic regression.

**Results:**

Higher educational attainment was significantly associated with better glycemic control (*p* = 0.015), lower BMI (*p* = 0.019), and greater nutritional literacy (*p* < 0.001). Participants who used ingredient lists had better glycemic control (*p* = 0.040), whereas exposure to incorrect nutritional information (*p* = 0.003) and difficulty accessing nutritional information (*p* = 0.011) were associated with poorer glycemic control. Overall, 51.2% of participants were obese, 38.7% had very poor glycemic control (HbA1c > 8%), and only 5.7% correctly interpreted nutrition facts. These findings suggest that nutrition literacy-related knowledge and behaviors may play an important role in glycemic control and obesity among adults with T2DM.

**Conclusion:**

Limited Nutrition literacy-related behaviors were associated with poorer diabetes-related outcomes. Targeted nutritional education may improve glycemic control and obesity management among adults with T2DM in Bahrain. Future longitudinal studies are needed to confirm these findings.

## 1 Introduction

Type 2 Diabetes Mellitus (T2DM) is one of the most significant and rapidly increasing chronic health problems worldwide, imposing a substantial burden on both individuals and healthcare systems (1). Globally, the prevalence of T2DM continues to rise due to sedentary lifestyles, unhealthy dietary habits, and aging populations, making it a major concern for public health policy and clinical practice. The Middle East is particularly affected, accounting for approximately 10% of the world’s diabetic population, with nearly half of these cases remaining undiagnosed, highlighting the silent yet escalating nature of the disease in the region (2). In Bahrain, T2DM has emerged as a critical public health issue, with a prevalence rate of 14.3% among adults, reflecting the broader global trend and underscoring the urgent need for effective prevention and management strategies tailored to the local context (3). Among the different factors influencing diabetes outcomes, nutritional literacy has gained increasing attention as a pivotal determinant of patients’ ability to manage their disease. Nutritional literacy, defined as the ability to seek, comprehend, and apply basic nutrition information, enables individuals to understand food labels, assess the nutritional quality of meals, and implement dietary recommendations to make informed food choices (4). This competency plays a central role in promoting overall health and well-being, but it is especially critical in chronic disease management where daily dietary decisions directly influence disease progression and outcomes. In diabetes management, nutritional literacy supports self-regulation of carbohydrate intake, portion control, and monitoring of food quality to maintain stable blood glucose levels. By understanding the effects of macronutrients and micronutrients on glycemic control, individuals with T2DM can make informed choices that minimize glucose fluctuations and reduce the risk of acute and long-term complications (5). Despite the increasing availability of nutritional information and food labeling systems, many people face difficulties in effectively interpreting and applying this knowledge in daily life due to limited literacy, numeracy, and health literacy skills, thereby diminishing the potential benefits of nutrition education (6).

In addition to its role in glycemic management, nutritional literacy is strongly linked to obesity, a major modifiable risk factor for T2DM and a central target in diabetes prevention strategies. Evidence suggests that low nutritional literacy is associated with poor food choices, excessive caloric intake, and increased risk of obesity, whereas higher nutritional literacy facilitates healthier dietary practices that support weight maintenance or loss (7). Excess body fat, particularly visceral adiposity, is a key driver of insulin resistance, one of the primary mechanisms underlying the pathophysiology of T2DM. Obesity-induced metabolic dysfunction and low-grade systemic inflammation further accelerate the risk of developing diabetes, making obesity reduction an essential component of prevention and management (8). The strong and bidirectional link between obesity and diabetes has been so well documented that the term “diabesity” has been adopted to highlight their interconnectedness (9). Importantly, improving nutritional literacy may offer an effective, sustainable approach for tackling both obesity and diabetes simultaneously, by equipping individuals with the knowledge and skills necessary to adopt healthier eating patterns. Conversely, deficits in nutritional knowledge and skills increase susceptibility to poor dietary behaviors and excess weight gain, reinforcing the cycle of obesity and T2DM progression (10).

Another cornerstone of diabetes management is achieving and maintaining optimal glycemic control, with glycated hemoglobin (HbA1c) serving as the standard biomarker of long-term glucose regulation. Clinical guidelines recommend maintaining HbA1c levels below 7% to reduce the risk of both microvascular and macrovascular complications associated with diabetes (11). Research consistently demonstrates that individuals with higher levels of nutritional literacy are more likely to achieve better glycemic control, as they can effectively apply nutrition knowledge to meal planning, food selection, and adherence to dietary recommendations. By empowering patients to proactively manage their diet, nutritional literacy reduces reliance on pharmacological interventions, enhances treatment adherence, and contributes to improved quality of life (12). This relationship emphasizes the importance of integrating nutritional literacy assessment and education into routine diabetes care as a means of optimizing disease outcomes.

Despite the evidence supporting the importance of nutritional literacy in diabetes management, significant gaps remain in understanding its role among Middle Eastern populations. Previous studies have largely focused on specific dietary interventions or nutrition education programs, with less attention to nutrition literacy-related knowledge and behaviors as potential determinants of diabetes outcomes (13). In Bahrain, where the prevalence of T2DM among adults is approximately 14.3%, limited nutritional literacy may have important implications for diabetes self-management and long-term health outcomes. Difficulties in obtaining, understanding, and applying nutrition information may reduce adherence to dietary recommendations and contribute to poor glycemic control and obesity. Poor glycemic control and obesity are, in turn, associated with an increased risk of diabetes-related complications and greater healthcare needs (14). Regional evidence has documented a substantial burden of diabetes-related complications among adults with T2DM, highlighting the clinical consequences of inadequate diabetes management (15). These consequences may also contribute to increased healthcare utilization and economic burden. However, evidence examining the relationship between nutrition literacy-related knowledge and behaviors, glycemic control, and obesity among adults with T2DM in Bahrain remains limited. This represents an important knowledge gap and a missed opportunity to develop culturally tailored interventions that address the dietary practices, food environment, and social factors influencing diabetes self-management. In Bahrain, where rapid economic and lifestyle changes have increased exposure to calorie-dense, nutrient-poor foods, understanding these factors is particularly important for informing effective diabetes prevention and management strategies.

In response to this gap, the present study aimed to assess nutritional literacy among patients with type 2 diabetes mellitus (T2DM) in Bahrain and to examine its association with glycemic control and obesity. Specifically, the study assessed three key domains: nutritional literacy, barriers to healthy eating, and blood sugar monitoring, and examined their relationships with glycemic control (HbA1c) and obesity. By generating evidence on these associations, the study seeks to provide insights to inform the development of targeted, culturally appropriate educational interventions and public health policies aimed at improving nutritional literacy and diabetes self-management. Ultimately, strengthening nutritional literacy may contribute to healthier dietary behaviors, improved glycemic control, better weight management, and a reduced burden of T2DM in Bahrain.

## 2 Methods

### 2.1 Study design

This cross-sectional study was conducted in July and August 2024 through a face-to-face interview administered as a questionnaire-based survey.

### 2.2 Setting and participants

This cross-sectional study was conducted across ten public primary healthcare centers in Bahrain, which were purposively selected to represent diverse geographic locations and patient demographics. These centers serve as the primary access points for adults with type 2 diabetes mellitus (T2DM) receiving care through non-communicable disease and diabetes clinics under the Ministry of Health. The sample size was determined using the single population proportion formula $\;\frac{Z_{\times P(1-P)}^{2}}{E^{2}}$ where $Z_{1-\frac{\alpha }{2}=Z\;(0.975)=1.96}$ at 95% confidence level, *p* = 0.50 as the expected proportion of T2DM patients in Bahrain, and E = 0.05 as the margin of error. Based on this calculation, the required sample size was 384 participants, providing adequate statistical power to detect significant associations within the study population. Participants were recruited using a consecutive sampling technique. Adult patients with a confirmed diagnosis of T2DM who attended scheduled diabetes clinic appointments during the study period were consecutively approached and invited to participate until the study period ended. Recruitment was conducted between July and August 2024. No advertisements or flyers were used, and participants did not receive any financial compensation for participation. Eligible patients who agreed to participate provided informed consent before completing the study questionnaire.

### 2.3 Inclusion and exclusion criteria

The inclusion criteria of this study comprised Bahraini adults, aged 18 years and above, with a confirmed diagnosis of T2DM. Participants were recruited from selected primary healthcare centers and were required to provide informed consent. Individuals were excluded if they were non-Bahraini, diagnosed with type 1 diabetes mellitus, pregnant, or declined to participate.

### 2.4 Data collection and instruments

Data were collected through face-to-face interviews using a structured questionnaire administered by trained members of the research team who were not involved in the participants’ clinical care. The questionnaire was self-developed based on a review of the relevant literature and the objectives of the present study. Data were collected using a structured questionnaire consisting of several sections. The first section collected sociodemographic characteristics, including age, sex, educational attainment, and area of residence. The second section collected diabetes- and health-related information, including relevant clinical information, while HbA1c and BMI were obtained from participants’ medical records. The third section assessed nutritional literacy-related knowledge and behaviors, focusing on participants’ ability to access, understand, interpret, and apply nutrition-related information. Items addressed understanding of nutritional information, interpretation of food-related information, use of ingredient lists when making grocery choices, access to reliable nutritional information, exposure to incorrect or conflicting nutritional information, and practical or psychological barriers to obtaining and using nutritional information. Nutritional literacy was therefore operationalized as a set of specific knowledge and behavioral indicators rather than a single composite score. The questionnaire consisted primarily of structured closed-ended questions with yes/no, multiple-choice, and Likert-scale response options, depending on the domain being assessed. Before the main data collection, the questionnaire was pilot-tested with 12 patients to assess its clarity and internal consistency. The pilot study demonstrated acceptable reliability, with a Cronbach’s alpha coefficient of 0.78.

### 2.5 Data analysis

Data from 364 participants were included in the final analysis. Quantitative analysis was performed using both descriptive and inferential statistics to examine the relationship between nutritional literacy, food label comprehension, obesity, and glycemic control. Categorical variables were summarized as frequencies and percentages, while continuous variables were expressed as means with standard deviations. The primary outcome variables were nutritional literacy, glycemic control, and obesity, while independent variables included age, gender, geographical area, occupation, educational level, weight, and height. Participants were classified into three categories: good glycaemic control (HbA1c < 7.0%), poor (HbA1c 7.0–8.0%), and very poor glycaemic control (HbA1c > 8.0%), based on the glycaemic targets recommended by the American Diabetes Association (ADA) Standards of Care. Categorical data were visually presented using pie charts and cluster bar charts. Associations between categorical variables were assessed using Chi-square tests, and logistic regression analysis was employed to evaluate the relationship between independent variables and binary outcome measures. Statistical significance was defined at a *p*-value < 0.05. Data analysis was conducted using the Statistical Package for the Social Sciences (SPSS), version 29.

### 2.6 Ethical consideration

Approval was granted from the Research and Ethics Committee at the College of Medicine and Health Sciences of Arabian Gulf University and the Ministry of Health of the Kingdom of Bahrain (E11-PI-5-24), after sharing all the documents and information needed to make sure we are following the ethical rules. The data were saved in a password-protected computer to make sure all information is kept strictly confidential and hidden, and encoded anonymously to protect the participants’ confidentiality.

## 3 Results

The mean age of the participants was 59.9 ± 10.8 years, and 54.7% were female. A total of 42.9% had completed secondary education, while 25.0% held a bachelor’s degree. Additionally, 42.0% of participants resided in the Southern Governorate. Nearly half of the participants (46.7%) reported not engaging in any physical activity, whereas only 8.0% reported being physically active 5–6 times per week (Table 1).

**Table 1:** Socio-demographic characteristics of the participants (*n* = 364).

Sociodemographic Characteristics	Frequency *N*	Percentage %
Age group (Years) (Mean ± SD) = (59.92 ± 10.81)		
< 45	38	10.4
46–55	60	16.5
56–65	152	41.8
> 65	114	31.3
Gender		
Male	165	45.3
Female	199	54.7
Educational level		
Did not complete primary	30	8.2
Primary (6th grade)	57	15.7
Secondary	156	42.9
College/University	91	25
Postgraduate	30	8.2
Area		
Muharraq	77	21.2
Northern	78	21.4
Southern	153	42
Capital (Manama)	56	15.4
Physical activity		
None	170	46.7
1–2 a week	91	25
3–4 a week	44	12.1
5–6 a week	29	8
1–2 every day	30	8.2

### 3.1 Participant characteristics, nutritional literacy, and diabetes management practices

More than half of the participants were classified as obese (51.2%), while 14.1% had a normal weight. Regarding glycemic control, 38.7% had very poor control (HbA1c > 8%), whereas 31.3% achieved good control (HbA1c < 7%). Most participants recognized the importance of portion control for maintaining a healthy weight, and 84.1% acknowledged the association between overweight and chronic diseases. Nutritional literacy and related behaviors showed mixed patterns. Overall, 51.1% of participants reported reading nutrition labels, 54.7% sought nutrition information, and among those who sought information, 46.7% reported obtaining it from websites. Additionally, 52.2% reported difficulty understanding nutrition information, although 83.8% recognized the importance of nutrition labels in diabetes management. Overall, 58.0% of participants reported checking ingredient lists to avoid specific ingredients; among these participants, 85.3% reported doing so because of their diabetes. Furthermore, 69.2% of the total study sample reported modifying their dietary habits based on nutrition information. However, 54.7% experienced difficulty understanding the language used on food packaging, highlighting the need for clearer nutrition labeling. When asked to evaluate product healthiness and calorie content, 85.2% correctly identified the healthier product, whereas 49.2% incorrectly estimated the total calorie content of an entire package, and only 5.7% identified the correct value, indicating gaps in specific nutritional knowledge. Regarding blood glucose monitoring, 54.7% reported checking their blood glucose levels several times per day, 38.2% checked once daily, 36.3% reported no episodes of hyperglycemia, and 50.5% reported no episodes of hypoglycemia.

### 3.2 Association between sociodemographic characteristics and glycemic control

Regarding the association between glycemic control categories and sociodemographic characteristics of patients with type 2 diabetes, the results of the chi-square test (Table 2) revealed that there is a significant relationship between glycemic control and educational level (*p* = 0.015). Patients with higher levels of education demonstrated better glycemic management; specifically, the proportion of individuals with poor glycemic control (HbA1c > 8%) decreased, while the proportion with good control (HbA1c < 7%) increased as educational attainment rose. The highest rates of very poor glycemic control (HbA1c > 8%) were observed among uneducated patients (53.3%) and those with only primary education (45.6%). No significant associations were found between glycemic control and other factors, including age, gender, area of residence, or physical activity.

**Table 2:** Cross-tabulation of sociodemographic factors and glycemic control (HbA_1c_ levels) among patients with type 2 diabetes mellitus in Bahrain.

Sociodemographic Characteristics	Good^a^ *n* (%)	Poor^b^ *n* (%)	Very poor^c^ *n* (%)	*p* value
Age (Years)				
<45	15 (39.4)	8 (21.1)	15 (39.5)	0.726
45–55	19 (31.7)	18 (30)	23 (38.3)	
56–65	44 (28.9)	52 (34.2)	56 (36.9)	
>65	36 (31.6)	31 (27.2)	47 (41.2)	
Gender				
Male	53 (32.1)	50 (30.3)	62 (37.6)	0.914
Female	61 (30.7)	59 (29.6)	79 (39.7)	
Educational level				
Uneducated	9 (30)	5 (16.7)	16 (53.3)	0.015
Primary	12 (21.1)	19 (33.3)	26 (45.6)	
Secondary	41 (26.2)	50 (32.1)	65 (41.7)	
College/University	36 (39.5)	27 (29.7)	28 (30.8)	
Postgraduate	16 (53.3)	8 (26.7)	6 (20)	
Area				
Muharraq	22 (28.5)	26 (33.8)	29 (37.7)	0.303
Northern	32 (41)	23 (29.5)	23 (29.5)	
Southern	41 (26.8)	44 (28.8)	68 (44.4)	
Capital	19 (33.9)	16 (28.6)	21 (37.5)	
Physical activity				
None	47 (27.7)	48 (28.2)	75 (44.1)	0.269
1–2/week	32 (35.1)	28 (30.8)	31 (34.1)	
3–4/week	13 (29.6)	17 (38.6)	14 (31.8)	
5–6/week	14 (48.3)	8 (27.6)	7 (24.1)	
1–2/day	8 (26.7)	8 (26.7)	14 (46.6)	

^a^Good glycemic control (HbA1c < 7.0%).

^b^Poor glycemic control (HbA1c 7.0–8.0%).

^c^Very poor glycemic control (HbA1c > 8.0%).

Values are presented as *n* (%). *p*-values were calculated using Pearson’s chi-square test to compare the distribution of participants across the three glycemic control categories (good, poor, and very poor) according to each sociodemographic and nutritional literacy variable. Statistical significance was set at *p* < 0.05.

### 3.3 Association between sociodemographic characteristics and BMI

The results of the chi-square test (Table 3) showed that there is a statistically significant relationship between BMI and gender, education, and physical activity. Gender showed a strong association (*p* = 0.004), with females exhibiting a higher prevalence of obesity (58.6%) compared to males (42.4%). Educational level was also significantly associated with BMI (*p* = 0.019), with obesity rates decreasing as education level increased. The highest prevalence of obesity was found among uneducated participants (73.3%), while the lowest was observed among those with postgraduate education (36.7%). Physical activity demonstrated a significant association with BMI (*p* = 0.026). Participants who were physically inactive had the highest obesity rate (60.4%), whereas those engaging in regular activity (1–2 times daily) reported a lower prevalence (50%). Although not statistically significant, the age group 45–55 years showed the highest obesity prevalence (58.3%), and the Southern governorate recorded the highest obesity rates among regions (53.6%).

**Table 3:** Cross-tabulation of sociodemographic characteristics by body mass index among patients with T2DM in Bahrain.

Sociodemographic Characteristics	Normal^a^ *n* (%)	Overweight^b^ *n* (%)	Obesity^c^ *n* (%)	*p* value
Age				
<45	8 (21.1)	12 (31.6)	18 (47.3)	0.546
45–55	9 (15.0)	16 (26.7)	35 (58.3)	
56–65	17 (11.3)	55 (36.4)	79 (52.3)	
>65	17 (14.9)	43 (37.7)	54 (47.4)	
Gender				
Male	31 (18.8)	64 (38.8)	70 (42.4)	0.004
Female	20 (10.1)	62 (31.3)	116 (58.6)	
Educational level				
Uneducated	3 (10.0)	5 (16.7)	22 (73.3)	0.019
Primary	6 (10.7)	15 (26.8)	35 (62.5)	
Secondary	17 (10.9)	58 (37.2)	81 (51.9)	
College/University	18 (19.8)	36 (39.5)	37 (40.7)	
Postgraduate	7 (23.3)	12 (40.0)	11 (36.7)	
Area				
Muharraq	10 (13.0)	26 (33.8)	41 (53.2)	0.412
Northen	15 (19.2)	32 (41.0)	31 (39.8)	
Southern	19 (12.4)	52 (34.0)	82 (53.6)	
Capital	7 (12.7)	16 (29.1)	32 (58.2)	
Physical activity				
None	23 (13.6)	44 (26.0)	102 (60.4)	0.026
1-2/week	17 (18.6)	37 (40.7)	37 (40.7)	
3-4/week	4 (9.1)	22 (50.0)	18 (40.9)	
5-6/week	5 (17.2)	10 (34.5)	14 (48.3)	
1-2/day	2 (6.7)	13 (43.3)	15 (50.0)	

^a^Normal weight (<25 kg/m^2^).

^b^Overweight (25.0–29.9 kg/m^2^).

^c^Obesity (≥30.0 kg/m^2^).

### 3.4 Associations between nutrition literacy, barriers to healthy eating, glycemic control, and BMI/obesity

The results of the chi-square test (Table 4) revealed that there is a significant relationship between grocery decision-making based on ingredient lists and glycemic control (*p* = 0.040). Patients who considered ingredient lists when shopping achieved better glycemic control, with 39.6% maintaining HbA1c < 7%, compared to only 23.5% among those who did not. Conversely, a higher proportion of participants who ignored ingredient lists had very poor glycemic control (42%) compared to those who used them (32.4%).

**Table 4:** Cross tabulation of nutrition literacy, barriers to healthy eating, and blood sugar monitoring with glycemic control (HbA1c levels) among patients with T2DM in Bahrain.

Nutrition and Health Determinants	Total	Glycemic Control (HbA1c Levels)			*p* value
		Good *n* (%)	Poor *n* (%)	Very poor *n* (%)	
Nutrition literacy					
Need help to understand the nutritional information					
Yes	190 (52.2)	58 (30.5)	51 (26.9)	81 (42.6)	0.233
No	174 (47.8)	56 (32.2)	58 (33.3)	60 (34.5)	
Make grocery choices based on the ingredients*					
Yes	182 (69.2)	72 (39.6)	51 (28)	59 (32.4)	0.040
No	81 (30.8)	19 (23.5)	28 (34.5)	34 (42)	
Influence of food design and packaging					
Yes	252 (69.2)	88 (34.9)	72 (28.6)	92 (36.5)	0.084
No	112 (30.8)	26 (23.2)	37 (33)	49 (43.8)	
Calorie content in the whole container					
I do not know	164 (45.1)	45 (27.4)	49 (29.9)	70 (42.7)	0.247
True knowledge	21 (5.8)	9 (42.8)	3 (14.3)	9 (42.9)	
Wrong knowledge	179 (49.2)	60 (33.5)	57 (31.9)	62 (34.6)	
Barriers to healthy eating					
Scared to change					
Yes	125 (43.3)	40 (32)	35 (28)	50 (40)	0.841
No	239 (65.7)	74 (31)	74 (31)	91 (38)	
Not getting support					
Yes	79 (21.7)	23 (29.1)	22 (27.9)	34 (43)	0.675
No	285 (78.3)	91 (32)	87 (30.5)	107 (37.5)	
Wrong information					
Yes	174 (47.8)	67 (38.5)	39 (22.4)	68 (39.1)	0.003
No	190 (52.2)	47 (24.7)	70 (36.9)	73 (38.4)	
Feeling stressed (Too much information is stressful)					
Yes	132 (36.3)	41 (31.1)	37 (28)	54 (40.9)	0.775
No	232 (63.7)	73 (31.5)	72 (31)	87 (37.5)	
Hard to find information					
Yes	135 (37.1)	50 (37)	28 (20.8)	57 (42.2)	0.011
No	229 (62.9)	64 (27.9)	81 (35.4)	84 (36.7)	
Small font size					
Yes	283 (77.7)	91 (32.2)	77 (27.2)	115 (40.6)	0.098
No	81 (22.3)	23 (28.4)	32 (39.5)	26 (32.1)	
Satisfaction with the flexibility in diet (time/type)					
Very dissatisfied	16 (4.4)	4 (25)	5 (31.3)	7 (43.7)	0.737
Dissatisfied	41 (11.3)	12 (29.3)	10 (24.4)	19 (46.3)	
Neutral	126 (34.6)	39 (31)	38 (30.2)	49 (38.8)	
Satisfied	88 (24.2)	33 (37.5)	22 (25)	33 (37.5)	
Very satisfied	93 (25.5)	26 (28)	34 (36.5)	33 (35.5)	
Blood sugar monitoring					
Monitoring blood sugar levels throughout the day					
Yes	199 (54.7)	55 (27.6)	65 (32.7)	79 (39.7)	0.214
No	165 (45.3)	59 (35.7)	44 (26.7)	62 (37.6)	
The frequency of checking blood sugar levels					
Not checking	39 (16.4)	15 (38.5)	10 (25.6)	14 (35.9)	0.227
1 Time	91 (38.2)	31 (34.1)	27 (29.6)	33 (36.3)	
2 Times	53 (22.3)	10 (18.8)	17 (32.1)	26 (49.1)	
3 Times	55 (23.1)	11 (20)	20 (36.4)	24 (43.6)	
Episodes of high blood sugar					
Not checking	63 (17.3)	31 (49.2)	16 (25.4)	16 (25.4)	<0.001
0	132 (36.3)	54 (40.9)	43 (32.6)	35 (26.5)	
1–2	102 (28)	18 (17.6)	36 (35.3)	48 (47.1)	
More than 2	67 (18.4)	11 (16.4)	14 (20.9)	42 (62.7)	
Episodes of low blood sugar					
Not checking	64 (17.6)	33 (51.6)	16 (25)	15 (23.4)	<0.001
0	184 (50.5)	55 (29.9)	50 (27.2)	79 (42.9)	
1–2	87 (23.9)	23 (26.4)	33 (38)	31 (35.6)	
More than 2	29 (8)	3 (10.3)	10 (34.5)	16 (55.2)	

*The item was a follow-up question answered only by participants who reported checking ingredient lists (*n* = 263); therefore, percentages for this item are based on this subgroup.

Additional significant associations were observed. Participants who reported receiving incorrect or conflicting nutrition information had poorer glycemic control than those who did not (*p* = 0.003). In addition, those who experienced difficulty finding reliable nutrition information were more likely to have poor glycemic control (*p* = 0.011). Other factors, such as the influence of food design and packaging (*p* = 0.084) and knowledge of calorie content (*p* = 0.247), did not show statistically significant associations. These findings highlight the importance of nutrition literacy, particularly informed grocery choices and access to reliable information in supporting effective glycemic management.

Regarding the association between Nutritional Literacy and BMI/Obesity, the chi-square analysis revealed a significant relationship between BMI categories and glycemic control outcomes (χ^2^ = 10.802, df = 4, *p* = 0.029). Obese participants accounted for more than half of the study sample (51.2%) and were disproportionately represented among those with very poor glycemic control (57.4%). In contrast, individuals with normal BMI were more likely to achieve good glycemic control (16.8%) and had the lowest proportion of very poor control (9.2%). Overweight participants showed intermediate patterns, with 33.3% falling into the very poor glycemic control category.

### 3.5 Nutritional knowledge across educational levels

The results of the Chi-Square test (Figure 1) show that nutritional knowledge differed significantly by educational level (χ^2^ = 36.104, df = 8, *n* = 364, *p* < 0.001). Overall, 49.2% of participants demonstrated incorrect nutritional knowledge, 45.1% reported that they did not know the correct answer, and only 13.3% answered correctly. Participants with postgraduate education had the highest proportion of correct responses, whereas the majority of uneducated participants reported that they did not know the correct answer.

**Figure 1:**
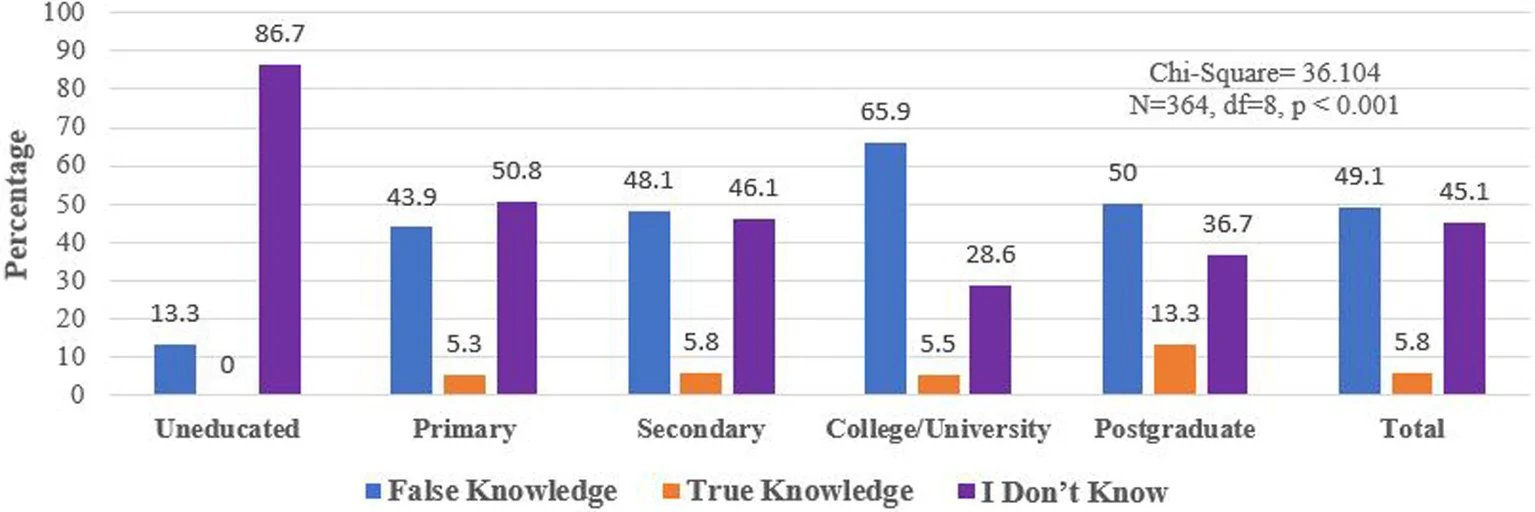
Percentage of participants answering the question: “How many calories are in the food/product,” according to educational level.

### 3.6 Barriers preventing patients from seeking nutritional information

The findings (Figure 2) highlight several key barriers preventing patients with type 2 diabetes mellitus (T2DM) from seeking or using nutritional information. More than three-quarters of participants reported that nutrition information on food labels or educational materials was too small to read, while nearly half indicated they often encountered inaccurate or misleading information, likely stemming from unreliable sources such as social media, non-professional advice, or poorly labeled products. Additionally, over one-third of patients reported difficulty accessing or locating nutritional information, and a similar proportion felt overwhelmed or stressed when trying to process it. Furthermore, about one-fifth of participants identified a lack of support from family, friends, or healthcare professionals as a barrier.

**Figure 2:**
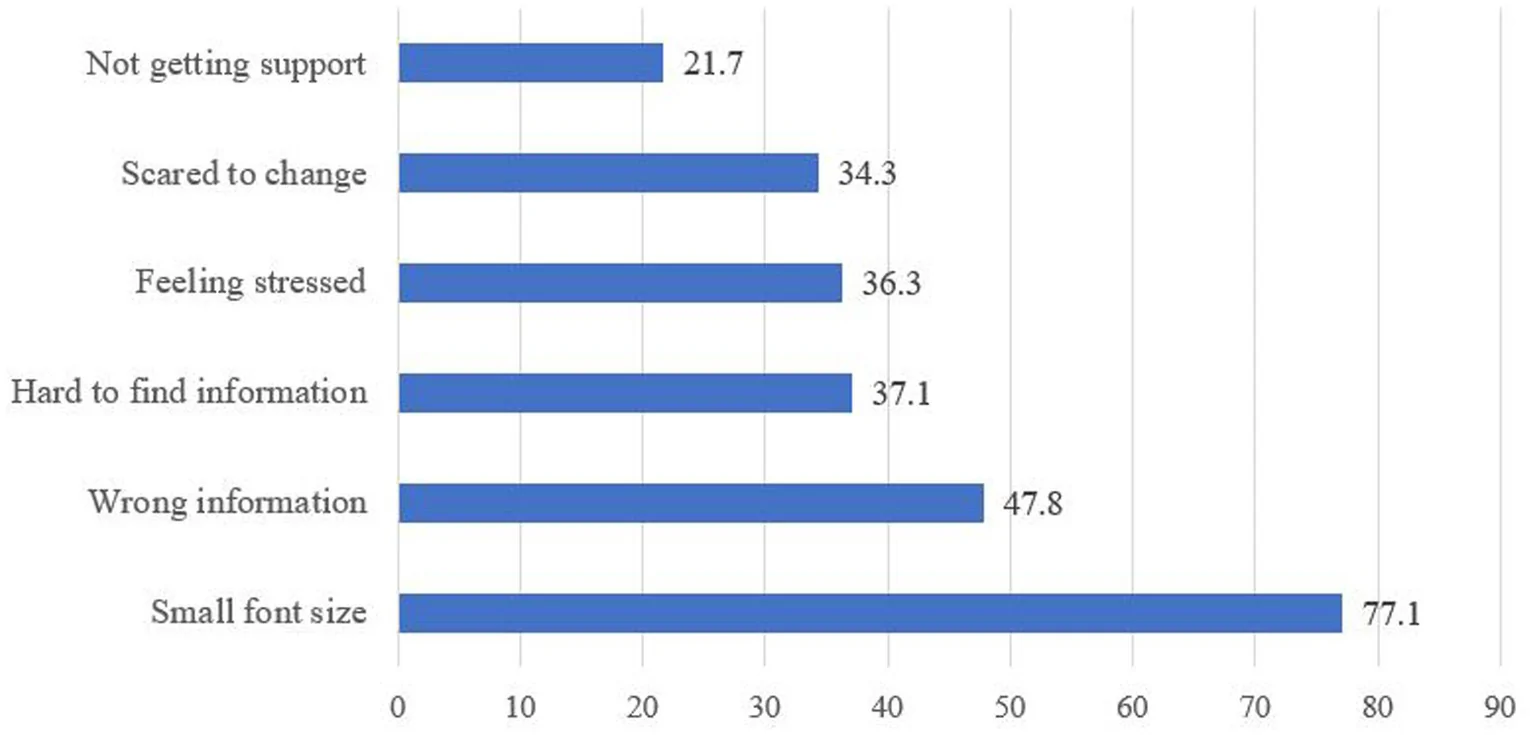
Percentage of participants answering “Yes” to each barrier to obtaining nutritional information. *N* = 364. Percentages represent the proportion of participants who answered “Yes” to each barrier item. Multiple responses were permitted; therefore, percentages do not sum to 100%.

The results of the univariate and multivariate logistic regression analyses provide important insights into the predictors of poor glycemic control among patients with type 2 diabetes mellitus (T2DM) in Bahrain (Table 5; Figure 3). Overall, most sociodemographic and lifestyle variables, including gender, age, physical activity, and area of residence, were not significantly associated with poor glycemic control after adjustment for confounders. Specifically, gender showed no significant effect, with males having similar odds of poor glycemic control compared to females in both crude and adjusted models. Similarly, different age categories did not demonstrate statistically significant associations, indicating that glycemic control was relatively consistent across age groups in this sample.

**Table 5:** Univariate and multivariate logistic regression predicting poor glycemic control among patients with T2DM in Bahrain (*n* = 364).

Characteristics	Crude odds ratio		Adjusted odds ratio	
	OR (95% C. I)	*p* value	OR (95% C. I)	*p* value
Gender				
Male	0.934 (0.599–1.457)	0.764	1.051 (0.631–1.750)	0.849
Female	Ref.		Ref.	
Age (years)				
< 45	0.708 (0.331–1.515)	0.373	0.734 (0.324–1.663)	0.458
45–55	0.996 (0.509–1.951)	0.991	1.063 (0.517–2.184)	0.868
56–65	1.133 (0.668–1.921)	0.643	1.299 (0.734–2.297)	0.369
> 65	Ref.		Ref.	
Level of education				
Uneducated	2.667 (0.924–7.699)	0.07	2.941 (0.924–9.368)	0.068
Primary	4.286 (1.642–11.183)	0.003	4.664 (1.665–13.067)	0.003
Secondary	3.206 (1.439–7.141)	0.004	3.498 (1.514–8.081)	0.003
College/University	1.746 (0.760–4.009)	0.189	1.795 (0.756–4.260)	0.185
Postgraduate	Ref.		Ref.	
Physical activity				
None	0.952 (0.396–2.286)	0.912	0.877 (0.344–2.236)	0.784
1–2 weeks	0.670 (0.268–1.676)	0.393	0.742 (0.280–1.963)	0.547
3–4 weeks	0.867 (0.308–2.445)	0.787	1.04 (0.351–3.079)	0.943
5–6 weeks	0.390 (0.131–1.157)	0.090	0.333 (0.105–1.052)	0.061
1–2 days	Ref.		Ref.	
Area				
Muharaq	1.284 (0.611–2.696)	0.509	1.50 (0.660–3.407)	0.333
Northern	0.738 (0.362–1.507)	0.405	0.812 (0.373–1.765)	0.599
Southern	1.403 (0.726–2.711)	0.314	1.535 (0.739–3.186)	0.250
Capital	Ref.		Ref.	
BMI class				
Normal	0.553 (0.287–1.069)	0.078	0.767 (0.377–1.557)	0.462
Overweight	0.534 (0.327–0.872)	0.012	0.587 (0.346–0.996)	0.048
Obese	Ref.		Ref.	

**Figure 3:**
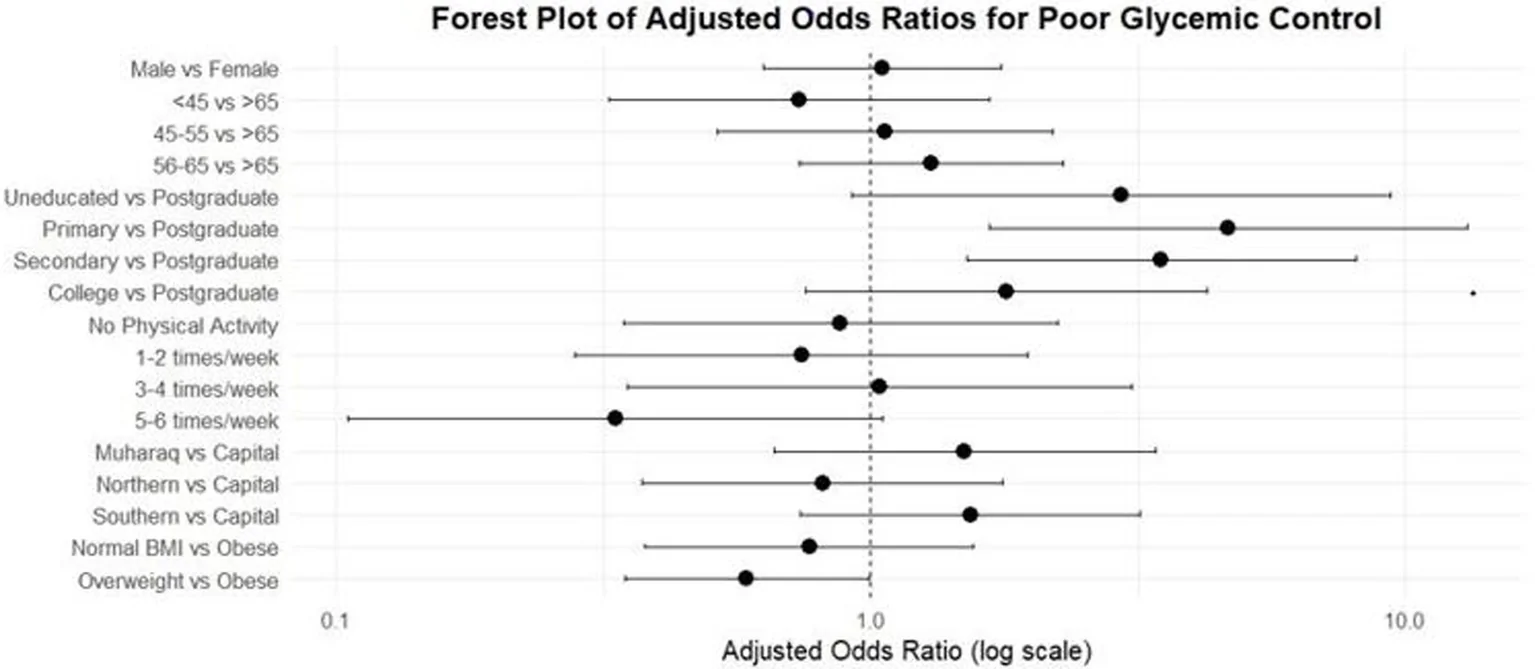
Forest plot of adjusted odds ratios (AORs) and 95% confidence intervals for factors associated with poor glycemic control among patients with type 2 diabetes mellitus in Bahrain. The points (dots) represent adjusted odds ratios (AORs), and horizontal lines represent 95% confidence intervals (CIs).

In contrast, the level of education emerged as an important factor. Patients with lower educational attainment, particularly those with primary and secondary education, had significantly higher odds of poor glycemic control compared to those with postgraduate education. In the adjusted model, individuals with primary education (AOR = 4.664, *p* = 0.003) and secondary education (AOR = 3.498, *p* = 0.003) were at markedly increased risk, suggesting that lower educational levels may be linked to limited nutritional literacy and poorer diabetes self-management.

Regarding physical activity, none of the categories showed statistically significant associations with glycemic control in the adjusted model, although there was a borderline trend suggesting that more frequent activity (5–6 times per week) may be associated with better glycemic outcomes. Area of residence was also not significantly associated with glycemic control, indicating relatively uniform outcomes across geographic regions.

Body mass index (BMI) showed a notable association. Overweight individuals had significantly lower odds of poor glycemic control compared to obese individuals (AOR = 0.587, *p* = 0.048). However, the normal BMI category did not reach statistical significance after adjustment.

## 4 Discussion and conclusion

Educational attainment emerged as an important independent predictor of poor glycemic control in this study. Participants with higher levels of education demonstrated better glycemic control, while those with only primary education had approximately 4.6-fold higher adjusted odds of poor glycemic control compared with participants with postgraduate education. This finding suggests that educational attainment may influence diabetes self-management through patients’ ability to understand health information, follow medical advice, and implement recommended lifestyle and dietary practices. Lower educational attainment is often associated with limited health literacy, which may impair patients’ ability to understand medical advice, interpret nutritional information, and implement dietary recommendations, ultimately contributing to poorer glycemic outcomes (16). This interpretation is consistent with evidence showing that patients with limited health literacy have significantly higher HbA1c levels because of difficulties in understanding medical and self-management instructions (16). In addition, a systematic review and meta-analysis reported an inverse association between socioeconomic status, including educational level, and HbA1c among people with type 2 diabetes, further supporting the present finding that lower educational attainment is associated with poorer glycemic control (17).

Importantly, the present study also identified substantial gaps in nutrition literacy-related knowledge and behaviors. Although nutritional literacy was not evaluated as an overall independent predictor in the multivariable model, several nutrition literacy-related indicators demonstrated significant associations with glycemic control in the bivariable analysis. Specifically, considering ingredient lists when shopping was associated with better glycemic control, whereas exposure to incorrect or conflicting nutritional information and difficulty finding reliable nutritional information were associated with poorer glycemic control. These findings suggest that the ability to obtain, interpret, and use nutrition-related information in everyday situations may be relevant to diabetes self-management. However, because an overall nutritional literacy measure was not included in the adjusted regression model, the present study cannot determine whether nutritional literacy is independently associated with glycemic control after accounting for educational attainment. Therefore, these findings should be interpreted as evidence of associations between specific nutrition literacy-related behaviors and glycemic control rather than as evidence of an independent effect of nutritional literacy (18).

The relationship between educational attainment and nutritional knowledge provides further context for these findings. Nutritional knowledge differed significantly across educational levels, with participants with lower educational attainment more likely to report incorrect nutritional knowledge or uncertainty regarding the correct answer, whereas participants with postgraduate education demonstrated the highest proportion of correct responses. These findings suggest that formal education may be related to patients’ capacity to understand and interpret nutrition-related information required for effective diabetes self-management. Since food-label interpretation and informed dietary choices require basic literacy and numeracy skills, patients with lower educational attainment may face greater challenges in applying nutritional recommendations in everyday practice. This may partly explain the observed association between lower educational attainment and poorer glycemic control. Nevertheless, this interpretation should be considered cautiously, as the present cross-sectional analysis did not formally test whether nutritional literacy mediates the relationship between education and glycemic control. Future studies using validated composite measures of nutritional literacy and appropriate mediation or multivariable analyses are warranted to determine whether nutritional literacy contributes independently to glycemic control beyond the effect of formal education.

The findings also highlight several practical and psychological barriers to the effective use of nutritional information. Approximately one-third of participants reported feeling stressed by nutrition information or being reluctant to change their dietary habits. Although these factors were not significantly associated with glycemic control in the present study, they may represent important barriers to adopting and maintaining healthier eating behaviors. Previous research has shown that patients may experience information overload, uncertainty regarding conflicting dietary advice, and anxiety about changing long-established dietary habits. These barriers highlight the need for individualized nutrition counseling that simplifies dietary recommendations and supports gradual, sustainable behavioral change.

The findings can also be interpreted through the Health Belief Model (HBM) and the Theory of Planned Behavior (TPB). From an HBM perspective, practical difficulties reported by participants, particularly the small font size and complexity of food labels, may represent perceived barriers that reduce patients’ ability to access and apply nutrition information. Although small font size was not significantly associated with glycemic control, it was reported by 77.1% of participants, suggesting that label accessibility may limit engagement with nutrition information and potentially influence dietary decision-making. Similarly, fear of changing dietary habits and feeling stressed by nutrition information may reduce patients’ confidence in adopting and maintaining healthier behaviors. The TPB provides an additional perspective by emphasizing behavioral beliefs, social influences, and perceived behavioral control in shaping health-related behaviors. Exposure to incorrect or unreliable nutritional information may influence beliefs about appropriate dietary practices, while difficulties in interpreting food labels may reduce perceived behavioral control over food choices. These findings suggest that diabetes nutrition interventions should extend beyond simply providing information and should also address practical and psychological barriers, strengthen patients’ confidence in applying nutrition information, and provide clear and reliable dietary guidance.

The observed findings regarding physical activity should be considered contextual rather than a primary finding of the study. Although physical activity was not independently associated with glycemic control in the adjusted analysis, physically inactive participants had a higher prevalence of obesity. This is consistent with evidence that reduced physical activity contributes to positive energy balance and increased adiposity through decreased energy expenditure (19). The absence of a significant independent association between physical activity and glycemic control may reflect differences in the intensity, duration, and consistency of physical activity among participants, as improvements in HbA1c may be more consistently achieved through structured and supervised exercise interventions than through general or inconsistent physical activity (20).

The discrepancy between the significant association with obesity and the lack of a clear direct relationship with glycemic control suggests that physical activity may operate indirectly. Because overweight individuals had lower odds of poor glycemic control than obese patients in this study, physical activity may influence glycemic outcomes primarily through its role in weight regulation. Prior evidence indicates that reductions in HbA1c associated with exercise are often accompanied by improvements in body composition (21), which supports this interpretation. Moreover, the cross-sectional design may have limited the ability to capture the cumulative effects of physical activity on HbA1c, a marker that reflects long-term glucose exposure. Variability in the intensity, duration, and consistency of physical activity may also have attenuated the observed association. Overall, these findings suggest that while physical inactivity is clearly associated with obesity, its relationship with glycemic control is more complex and may be mediated by body mass index.

The relationship between obesity and glycemic control in type 2 diabetes is well established. These findings suggest that obesity is closely linked to both poorer glycemic outcomes and lower nutritional literacy-related behaviors, as obese participants were less likely to use ingredient lists and more likely to report difficulty accessing or interpreting nutrition information. Together, these results reinforce the role of nutritional literacy not only in glycemic regulation but also in weight management among adults with type 2 diabetes. In this study, overweight participants had significantly lower odds of poor glycemic control compared with obese participants (AOR = 0.587, *p* = 0.048), indicating that increasing adiposity is associated with worsening glycemic control. This may be explained by the role of excess adipose tissue in promoting insulin resistance, chronic inflammation, and impaired insulin signaling, all of which contribute to metabolic dysregulation. The observed difference between overweight and obese individuals suggests that even moderate increases in adiposity may adversely affect glucose regulation. This is consistent with the American Diabetes Association, which states that a modest weight reduction of 3–7% can produce clinically meaningful improvements in glycemic control (22). Similarly, previous studies have shown that weight loss in overweight and obese patients with T2DM improves glycemic outcomes, whereas higher body weight is associated with poorer glucose regulation (23).

The so-called obesity paradox, in which overweight individuals with T2DM sometimes appear to have more favorable outcomes than those with normal weight, has been widely described. However, this pattern may be influenced by gender and population-specific factors. In the present study, overweight participants had lower odds of poor glycemic control than obese individuals, yet no significant association was observed between gender and glycemic control, despite a higher prevalence of obesity among females than males. This indicates that the apparent protective effect of overweight status did not translate into a gender-specific advantage in this population (24).

Biological differences between males and females, particularly in fat distribution, may partly explain gender-related variation in metabolic risk. Males typically accumulate more visceral fat, which is strongly associated with insulin resistance, whereas females generally have more subcutaneous fat, which may confer relatively lower metabolic risk (25). However, the lack of a significant gender difference in glycemic control in this study suggests that such biological mechanisms may be outweighed by other factors. Importantly, the findings indicate that educational level and nutritional literacy may play a more substantial role in shaping both obesity and glycemic outcomes among Bahraini patients with T2DM. Lower educational attainment was associated with higher BMI and poorer glycemic control, suggesting that the relationship between obesity and clinical outcomes is likely influenced by behavioral and knowledge-based factors rather than weight alone. This is consistent with evidence that the obesity paradox may partly reflect confounding by comorbidities, disease severity, and reverse causation, whereby unintentional weight loss in sicker individuals leads to worse outcomes among those with normal weight (26).

The relatively high prevalence of obesity in this cohort highlights the importance of contextual factors in Bahrain, including dietary patterns, lifestyle behaviors, and reliance on nutritional information. In this setting, limited nutritional literacy may reduce the ability to make appropriate dietary choices, thereby weakening any potential protective effect associated with overweight status. Accordingly, the obesity paradox observed in this study should be interpreted cautiously, as it likely reflects underlying socioeconomic and behavioral influences rather than a genuine physiological advantage of increased body weight. Overall, the interaction between gender, obesity, and glycemic control appears to be complex and more likely driven by modifiable factors such as education and nutritional literacy than by gender-specific biology alone.

A statistically significant association was also observed between using ingredient lists during grocery shopping and improved glycemic control, as reflected by lower HbA1c levels among patients with T2DM (*p* = 0.040). This finding is consistent with evidence that food-label education can improve diet quality and support better glucose control (27). Reading ingredient lists may help patients identify hidden sugars and ultra-processed components, thereby encouraging the selection of higher-fibre, lower-glycaemic index foods (28). Such foods are typically richer in fibre and whole grains, both of which have been associated with improved glycemic control. Supporting this, higher fibre intake has been inversely associated with HbA1c, and individuals with optimal glycemic control often consume more than 15 g/day of fibre and obtain around 40% of carbohydrates from whole grains (28).

The present study also identified a substantial nutritional literacy gap among Bahraini patients with T2DM, with only 5.7% correctly identifying calorie content. This finding highlights a major barrier to effective food-label interpretation and informed dietary decision-making. Notably, 38.7% of patients had poor glycemic outcomes (HbA1c > 8%), reinforcing the importance of nutritional literacy as a key component of diabetes self-management in Bahrain. Improving food-label comprehension within local healthcare settings may therefore help patients make more informed dietary decisions and achieve better long-term outcomes.

Findings from neighboring countries further reinforce the importance of nutritional literacy in diabetes care. Al-Marri et al. reported that patients with T2DM in Qatar who had higher nutritional literacy achieved better glycemic control, with food-label comprehension emerging as an important determinant of HbA1c outcomes (29). Similarly, Alshahrani et al. found that Saudi patients with diabetes had poor knowledge of food labels, which limited their ability to align dietary intake with clinical recommendations (30). These studies are consistent with the present findings and indicate that nutritional literacy is a shared challenge across Middle Eastern populations and an important target for intervention.

Evidence from other regions also supports these observations. A Brazilian analysis found that inadequate health literacy among T2DM patients ranged widely and was strongly associated with higher HbA1c levels and more complications (14). Likewise, a study from Turkey showed that nutritional literacy improves metabolic control and quality of life, underscoring the clinical relevance of food-label comprehension (31). Similarly, a Dutch study of patients with type 2 diabetes found that higher self-perceived food literacy was associated with significantly better diet quality, particularly greater intake of vegetables, fruit, legumes and fish, and lower intake of processed meat and sweetened beverages (32). Together, these findings confirm that nutritional literacy is not merely an academic construct but a practical skill that directly affects dietary quality and glycemic regulation.

Small font size on food packaging was the most frequently reported barrier to seeking nutritional information, identified by 77.1% of participants. Although this factor was not significantly associated with glycemic control (*p* = 0.098), its high prevalence suggests that it is a meaningful practical obstacle to label use in this population. This is consistent with prior research showing that label design features, particularly text size and visual clarity, strongly influence consumer engagement with nutritional information at the point of purchase (4). Cowburn and Stockley likewise reported that readability, presentation, and complexity are among the strongest predictors of whether consumers use nutrition labels, especially among older adults and those with lower literacy (33). Given the mean age of participants in this study, age-related visual decline may further amplify the impact of small print as a barrier.

The lack of a direct statistical association with glycemic control should not be interpreted as evidence that legibility is unimportant. Rather, small font size likely functions as an indirect barrier by reducing the likelihood that patients will use food labels at all, with its effect on glycemic outcomes operating through diminished label engagement. This interpretation is supported by Alshahrani et al., who found that difficulty reading nutrition labels was among the most commonly reported barriers among patients with diabetes, and only a small minority could read labels without difficulty (30). It is also consistent with the present finding that participants who used ingredient lists had significantly better glycemic control. Improving font size, contrast, and front-of-pack labelling, together with targeted nutrition education, may therefore improve label accessibility and support more informed dietary decisions among patients with T2DM. This study provides Bahrain-specific evidence on nutrition literacy-related knowledge and behaviors, food-label use, and barriers to obtaining and understanding nutrition information among adults with T2DM, and examines their relationships with glycemic control and obesity. Although previous studies from Qatar, Saudi Arabia, Turkey, Brazil, and the Netherlands have reported associations between nutrition or food literacy and diabetes-related outcomes, limited evidence has examined these relationships within the Bahraini primary healthcare context while also considering practical barriers to the use of nutrition information. The findings therefore provide context-specific evidence that may help inform culturally appropriate interventions in Bahrain, particularly the integration of nutrition counseling into routine primary diabetes care and efforts to improve the accessibility and clarity of food-label information.

However, several limitations should be acknowledged. The generalizability of the findings may be limited by the difficulty in accessing patients from private hospitals, where a substantial proportion of individuals with T2DM in Bahrain receive care. In addition, participants were not selected using simple random sampling, which may introduce selection bias and reduce representativeness. Finally, the relatively short data collection period made it challenging to achieve the desired sample size, which may have affected the precision and external validity of the results. Furthermore, nutrition literacy was assessed using individual questionnaire items rather than a validated composite score. The questionnaire was self-developed and demonstrated acceptable internal consistency (Cronbach’s α = 0.78); however, formal validity testing, such as content or construct validity assessment, was not performed. These limitations should be considered when interpreting the findings and highlight the need for future studies using validated nutritional literacy instruments and larger, more representative samples.

In conclusion, this study highlights the importance of nutrition literacy-related knowledge and behaviors in diabetes management among adults with type 2 diabetes in Bahrain. Future longitudinal studies using validated nutritional literacy instruments with established psychometric properties and larger samples are warranted to confirm these findings and evaluate the effectiveness of nutrition literacy interventions in improving glycemic control and reducing obesity-related complications (34).

## Data Availability

Publicly available datasets were analyzed in this study. This data can be found at: datasets are available on request: The raw data supporting the conclusions of this article will be made available by the authors, without undue reservation.
